# Case Report: A newly identified cause of ST-segment elevation in lead aVR

**DOI:** 10.3389/fcvm.2026.1782401

**Published:** 2026-04-29

**Authors:** Wei Li, Yang Liu, Pan Feng, Chaoji Huangfu, Dayong Du

**Affiliations:** 1 Department of Cardiology, The 305 Hospital of People's Liberation Army of China, Beijing, China; 2 Beijing Institute of Radiation Medicine, Beijing, China

**Keywords:** CABG, lead aVR, LIMA, ST-segment elevation, coronary-subclavian steal syndrome

## Abstract

**Background:**

ST-segment elevation in lead aVR typically signifies left main or triple-vessel disease. In post-coronary artery bypass grafting (CABG) patients, this pattern, alongside patent grafts, necessitates investigating alternative causes of myocardial ischemia.

**Case presentation:**

A 74-year-old male post-CABG presented with angina and ECG showing ST elevation in aVR with diffuse depression. Angiography revealed severe native coronary disease but patent grafts, including left internal mammary artery (LIMA) to left anterior descending artery. Persistent symptoms prompted further evaluation, which identified a critical left subclavian artery stenosis, compromising LIMA flow.

**Conclusion:**

This case identifies left subclavian artery stenosis as a cause for aVR ST elevation in post-CABG patients by limiting LIMA graft inflow. It emphasizes the importance of evaluating extracoronary vascular lesions in recurrent ischemia despite patent grafts. Recognition and endovascular treatment of this condition can rapidly resolve symptoms and ECG abnormalities.

## Introduction

Electrocardiographic ST-segment elevation in lead aVR, particularly when accompanied by widespread ST depression, is a well-established marker of extensive myocardial ischemia (1). It classically signals critical left main coronary artery (LMCA) disease or severe triple-vessel disease, necessitating immediate consideration for emergency revascularization (2). This case presents a rare and diagnostically challenging scenario where persistent ischemia and the characteristic aVR ST-segment elevation occurred despite angiographically confirmed patency of all bypass grafts.

This case is unique because it identifies a critical, extracoronary vascular lesion—severe left subclavian artery stenosis—as the definitive culprit. This stenosis functionally compromised the inflow to a patent left internal mammary artery (LIMA) graft, inducing a coronary-subclavian steal phenomenon and precipitating ischemia in the left anterior descending artery territory. Coronary-subclavian steal syndrome is an under-recognized entity, reported to complicate only 0.2% to 6.8% of cases where a LIMA graft is used. This case underscores that in post-CABG patients with otherwise unexplained ischemia, a high index of suspicion for proximal inflow disease is essential, and findings such as an inter-arm systolic blood pressure difference of >10 mmHg can provide a critical diagnostic clue.

## Case description

A 74-year-old man with a 4-year history of exertional chest tightness presented with progressive symptoms over four days, accompanied by shoulder and upper back pain. His medical history included coronary artery disease, type 2 diabetes mellitus, diabetic nephropathy, and end-stage renal disease requiring maintenance hemodialysis. Four years earlier, coronary angiography had revealed diffuse calcification and severe stenoses—50% in the left main coronary artery (LM), 95%–99% in the proximal left anterior descending artery (LAD), 90% in the proximal left circumflex artery (LCx), 90% in the proximal intermediate branch, and 85% in the proximal-to-mid right coronary artery (RCA). He subsequently underwent three-vessel coronary artery bypass grafting (CABG), including a left internal mammary artery (LIMA) graft to the mid-LAD, and saphenous vein grafts from the ascending aorta to both the intermediate branch and posterior descending artery (PDA).

**Figure 1:**
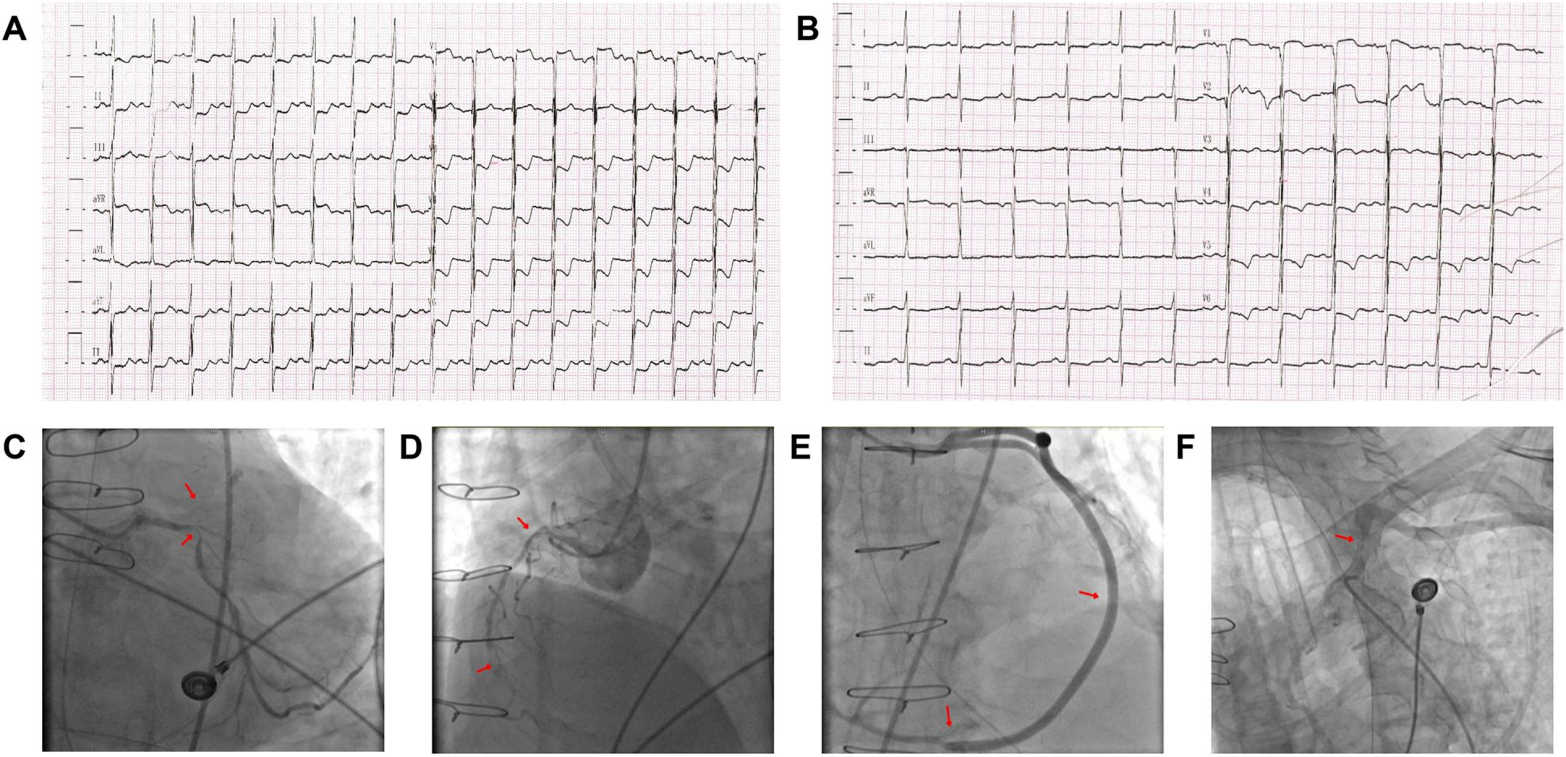
The results of the patient's electrocardiogram and angiography. **(A)** Electrocardiogram during an episode of angina demonstrating ST-segment elevation in lead aVR, with ST-segment depression in ≥8 leads and ST-segment elevation in leads aVR. **(B)** Post–left subclavian artery stent implantation electrocardiogram. The patient's symptoms resolved, and ST-segment elevation in lead aVR disappeared. **(C)** Coronary angiography demonstrating 50–60% diffuse stenosis with calcification of the left main coronary artery, proximal occlusion of the left anterior descending artery and intermediate branch, and 95% calcified stenosis in the proximal left circumflex artery. **(D)** Coronary angiography demonstrating approximately 90% diffuse stenosis with calcification in the proximal right coronary artery and distal occlusion. **(E)** Bypass graft angiography showing patent right coronary venous graft with no anastomotic stenosis. **(F)** Selective angiography of the left subclavian artery demonstrating severe proximal stenosis.

Initial laboratory testing revealed markedly elevated cardiac biomarkers: high-sensitivity troponin I 8,582.70 pg/mL, CK-MB 21.60 ng/mL, myoglobin 315.20 ng/mL, and BNP 1,526.8 pg/mL. Renal function was severely impaired (creatinine 693.0 *μ*mol/L; urea 19.90 mmol/L). The electrocardiogram (ECG) recorded during episodes of angina demonstrated ST-segment elevation in lead aVR and V1, accompanied by diffuse ST-segment depression in leads I, III, aVL, aVF, and V3–V6 (Figure 1A). Echocardiography demonstrated segmental wall motion abnormalities predominantly in the anterior and apical walls, consistent with the left anterior descending (LAD) coronary artery territory and compromised patency of the left internal mammary artery (LIMA) graft. Left ventricular ejection fraction was mildly reduced (32%), with mild left ventricular dilation (end-diastolic anteroposterior dimension 56 mm). Mild basal interventricular septal hypertrophy was noted, along with mild regurgitation of the mitral, tricuspid, and aortic valves.

The ECG demonstrated sinus rhythm with pronounced ST-segment elevation in lead aVR and V1, together with ST-segment depression in ≥8 leads—an ischemic pattern typically associated with acute left main coronary artery occlusion or severe triple-vessel disease. Although this pattern often warrants emergency revascularization, the patient's history of CABG and the absence of new severe stenosis of bypass grafts made native coronary occlusion an incomplete explanation for the persistent ECG abnormalities. This prompted further evaluation for alternative etiologies capable of causing diffuse subendocardial ischemia in the LAD territory despite patent bypass grafts.

Urgent coronary angiography revealed 50%–60% diffuse LM stenosis, chronic total occlusion of the proximal LAD and intermediate branch, and 95% calcified stenosis of the proximal LCx. The RCA showed diffuse 90% proximal stenosis with distal occlusion (Figures 1C–F). Notably, all bypass grafts—including the LIMA–LAD and both saphenous vein grafts—were patent without significant stenosis.

Given the severe LCx lesion, balloon angioplasty and stent implantation were performed. However, the patient continued to experience angina with persistent ST-segment elevation in lead aVR. Repeat review of angiographic imaging revealed a previously overlooked critical stenosis of the proximal left subclavian artery. This lesion had initially escaped detection because the angiographic catheter was advanced deeply into the LIMA origin, yielding the misleading impression that LIMA flow was normal.

Bedside reassessment revealed a weaker left radial pulse and a 30-mmHg systolic blood pressure difference between the upper extremities, supporting impaired LIMA perfusion due to proximal subclavian disease. Percutaneous transluminal angioplasty with stent implantation of the left subclavian artery was subsequently performed. Pre-stent angiography revealed a 95% diameter stenosis of the left subclavian artery. Pressure gradient measurements across the lesion demonstrated a 60 mmHg pressure drop (pre-stent) vs. 0 mmHg post-stent implantation, confirming hemodynamic significance. Quantitative LIMA flow data (e.g., fractional flow reserve, coronary flow reserve) was not measured during this procedure, as it was a routine percutaneous intervention without dedicated flow assessment. The patient's angina resolved immediately, and ST-segment elevation in lead aVR disappeared on follow-up ECGs (Figure 1B). No further ischemic symptoms occurred during rest or physical activity. Taken together, these findings indicate that severe left subclavian artery stenosis caused flow limitation to the LIMA graft, functionally mimicking LIMA occlusion and precipitating LAD-territory ischemia with characteristic aVR ST elevation. In this retrospective case report, a 3-month clinical follow-up was performed, during which the patient remained asymptomatic for angina and dyspnea, and duplex ultrasound confirmed no subclavian artery restenosis.

## Discussion

ST-segment elevation in lead aVR accompanied by diffuse ST depression is commonly interpreted as a marker of extensive subendocardial ischemia and is strongly associated with left main or triple-vessel disease (3). While ST elevation in aVR with diffuse ST depression often suggests proximal coronary disease, this pattern is non-specific and must be interpreted in context of hemodynamic status and exclusion of alternative causes. Critical differentials include severe anemia, acute hemodynamic instability, and demand ischemia in heart failure. In this case, stable hemodynamics (systolic BP >90 mmHg, heart rate 68 bpm) and hemoglobin level of 103 g/dL (within target range for dialysis patients: 9–11 g/dL) supported a coronary vascular etiology, ruling out major non-ischemic contributors.

The CABG was performed in April 2021. Preoperative imaging confirmed patent left subclavian artery without stenosis at that time. The current finding of left subclavian artery stenosis represents a newly diagnosed lesion. Post-CABG patients present a unique diagnostic challenge. When the LIMA is used as a conduit, proximal left subclavian artery stenosis may critically reduce LIMA flow, leading to the so-called coronary–subclavian steal phenomenon (4). This condition is underrecognized and may be overlooked during routine coronary angiography—particularly when catheter engagement directly enters the LIMA origin and masks proximal stenosis.

This case demonstrates several important diagnostic insights. Subclavian artery stenosis is an important differential diagnosis for persistent ischemia and ST elevation in aVR among post-CABG patients with LIMA grafts, especially when coronary grafts remain patent. Clinical signs such as inter-arm blood pressure differences, reduced radial pulse amplitude, or upper limb claudication may provide critical diagnostic clues. Invasive angiography may fail to detect subclavian stenosis if selective LIMA engagement bypasses the proximal segment; therefore, dedicated imaging of the subclavian artery (via angiography, duplex ultrasound, or CT angiography) is essential when ECG or symptoms are unexplained by graft status (5). Identification and treatment of subclavian stenosis often lead to rapid symptom resolution and normalization of ischemic ECG changes, as highlighted in this case. While comprehensive long-term data was not systematically collected due to the retrospective nature of this acute presentation case review, the immediate resolution of ST-elevation and ischemic symptoms post-stenting provided critical diagnostic corroboration.

The patient has multiple risk factors. However, the temporal relationship between the subclavian stenting and the complete resolution of ST-segment elevation and angina suggests that the subclavian stenosis was the primary driver of the acute episode. If the ischemia were solely due to diffuse native CAD or ESRD, stenting the subclavian artery would not have resulted such rapid normalization.

## Conclusion

This case expands the spectrum of etiologies associated with ST-segment elevation in lead aVR and emphasizes that extracoronary vascular lesions must be considered when evaluating recurrent ischemia in patients with previous CABG.

## Data Availability

The original contributions presented in the study are included in the article/Supplementary Material, further inquiries can be directed to the corresponding authors.
